# Component-Based Modelling for Scalable Smart City Systems Interoperability: A Case Study on Integrating Energy Demand Response Systems

**DOI:** 10.3390/s16111810

**Published:** 2016-10-28

**Authors:** Esther Palomar, Xiaohong Chen, Zhiming Liu, Sabita Maharjan, Jonathan Bowen

**Affiliations:** 1 School of Computing and Digital Technology, Birmingham City University, Birmingham B4 7XG, UK; 2 Department of Computer Science, University of Illinois at Urbana-Champaign, Champaign, IL 61801, USA; xc3@illinois.edu; 3 Centre for Research and Innovation in Software Engineering, Southwest University, Chongqing 400700, China; zhimingliu88@swu.edu.cn; 4 Networks Department, Simula Research Laboratory, Fornebu 1364, Norway; sabita@simula.no; 5 School of Engineering, London South Bank University, London SE1 0AA, UK; jonathan.bowen@lsbu.ac.uk

**Keywords:** smart city system modelling, component-based architecture design, component system interoperability and coordination, scalable modelling, cooperative demand response

## Abstract

Smart city systems embrace major challenges associated with climate change, energy efficiency, mobility and future services by embedding the virtual space into a complex cyber-physical system. Those systems are constantly evolving and scaling up, involving a wide range of integration among users, devices, utilities, public services and also policies. Modelling such complex dynamic systems’ architectures has always been essential for the development and application of techniques/tools to support design and deployment of integration of new components, as well as for the analysis, verification, simulation and testing to ensure trustworthiness. This article reports on the definition and implementation of a scalable component-based architecture that supports a cooperative energy demand response (DR) system coordinating energy usage between neighbouring households. The proposed architecture, called refinement of Cyber-Physical Component Systems (rCPCS), which extends the refinement calculus for component and object system (rCOS) modelling method, is implemented using Eclipse Extensible Coordination Tools (ECT), i.e., Reo coordination language. With rCPCS implementation in Reo, we specify the communication, synchronisation and co-operation amongst the heterogeneous components of the system assuring, by design scalability and the interoperability, correctness of component cooperation.

## 1. Introduction

Interoperability challenges of smart city systems can be overcome with an open architecture approach that facilitates the integration of devices and applications and enables seamless sharing of data between systems and reuse of code. In 2014, Palomar et al. [1] introduced an extension of the refinement calculus of component and object systems (rCOS) modelling method [2,3] that supports the development of a smart community demand response (DR) system [4,5]. A cooperative DR system is coordinating the energy usage among neighbouring households [6] making the overall consumption more sustainable and efficient. In a centralised scenario, an aggregator coordinates and optimises neighbourhood-level aggregated power demand, given the total hourly power consumption across neighbouring households, with the available supply from renewables at the utility sub-station. Both the cooperation of the consumers targeting the available renewable energy supply and the heterogeneity of the systems and devices involved motivate the need of a component based modelling method for the development of a novel smart community DR solution that tends to promote transformation of the whole energy value chain.

This article presents the implementation of the refinement of Cyber-Physical Component Systems (rCPCS) architecture using Reo Coordination Language [7] and Eclipse Extensible Coordination Tools (ECT) [8]. rCPCS captures the evolving nature of the system architecture and helps in dealing with the dynamically growing functional complexity of the proposed DR framework, which comprises a number of distributed, dynamic components deployed over large networks of heterogeneous platforms. Animation of the Reo model in Eclipse will demonstrate that the formal architecture matches its informal description and adequately describes the modelled system. In particular, our main aim is to show (1) how we design a system that is able to scale up easily with no remarkable change in its architecture, and (2) how we achieve “separation of concerns”; that is, we can focus on what a component does in terms of its interface behaviour ignoring the “how” at an early stage of system design process. Indeed, early validation of the interoperability and scalability of the complex and networked social-technical smart city system designs are key concerns among the technology solutions. Novel software architectures and developments that encounter these challenges are then desired to build correct and reliable smart city applications.

There is a need for a systematic and scalable method (i.e., modelling language with a semantic theory) for modelling, analysis and validation (sound techniques and tools for analysis, verification and simulation) that addresses the dynamically evolving nature of the applications within smart cities. In this regard, formal models and languages such as Reo capture the foundations of the system, building a common understanding between the system components and participants, and making it easy to design and analyse the dynamics of scalable systems and to automatically validate conformance and ensure interoperability. The Reo model is supporting the following essential information flows: (1) information flows between the utility and the aggregator; (2) information flows between each consumer and the aggregator and (3) information and control flows between a consumer and his appliances. We model the co-ordination between components leaving most of their functionalities, especially in the case of appliances, to be implemented in the future. However, we will show that our model is implementation-independent, so any possible implementation for the aggregator will work for instance. Both rCOS and Reo have been used in the design and verification of service-oriented and component-based software systems, but this paper gives a first attempt for them to be used in modelling Smart City systems as a Cyber-Physical Component System.

The rest of the paper is organised as follows. We outline related work in Section 2. Section 3 describes our cooperative DR framework, including roles and main phases. We present the cyber-physical component-based modelling rCPCS in Section 4 and the implementation details using Reo in Section 5. We end with a discussion in Section 6 and conclude in Section 7.

## 2. Related Work

The technological complexity, as well as the complexity of the various sectorial services involved within a smart city, has been addressed by a formal approach to open architectures and platforms seeking the creation of global ecosystems, ensuring the interoperability and the creation of standard data models [9]. Moreover, formal modelling has all the characteristics required to replace programming and offer higher productivity, refinement and important features by design.

Several software architectures have been proposed such as in [10,11,12,13], with different goals. Most architectures were designed for specific purposes like real-time monitoring, energy efficiency, distributed sensing and processing, mobility, or privacy purposes and, other requirements were eventually added in order to increase its adaptability and portability. For example, a middleware is implemented using OSGi (—The OSGi Alliance, formerly known as the Open Services Gateway initiative, is a worldwide consortium of technology innovators that advances a proven and mature process to create open specifications that enable the modular assembly of software built with Java technology—) bundles in [10] to deal with objects interoperability and heterogeneous information handling. An architecture, initially developed with the objective of providing a consistent model and interfacing standardisation for building Internet of Things (IoT) applications is adapted to the context of smart cities, providing the API for queries, and basic abstractions, such as events, states, and content management services. Also with a middleware shape, Filipponi et al. [11] developed an interoperable event-driven architecture through an Interoperability Open Platform (IOP) for the implementation of information services for monitoring public areas and infrastructures. The architecture formalises the interacting objects ecosystem (sensors, devices, appliances and embedded systems), and the services and custom processes. None of these proposals deals with system composition and/or coordination.

There is a great deal of work on formal models of component systems [2,7,8,14,15,16,17,18]. In particular, the modelling with relational calculus of object and component systems, rCOS [2], assists in the formal modelling of software architectures for complex and integrated information and networking systems, monitoring environments and collaborative workflows involving many different kinds of stakeholders and end users across different domains [19,20]. rCOS supports interoperable compositions of components that exhibit interacting behaviour with the environment and, for that, the local data functionality is implemented in different programming paradigms, including modular, procedural and object-oriented programming. Hence, in rCOS, we can deal not only with the interaction among components and processes, but also the state-based functional behaviour of components [3,21].

In Reo coordination language, [7] a channel-based model is used for describing coordination. Components are loosely coupled and can only communicate with each other via a channel, which also coordinates the behaviour of the components. Reo was originally designed to support the compositional construction of web services. In particular, work in [22,23] introduces a Reo coordination middleware to coordinate the interactions among application components by narrowing gaps between real-world applications and low-level hardware and software. Following this idea, Zlatev et al. [24] specify and implement in Reo the negotiation protocols for e-commerce that support compositional construction and dynamical reconfiguration. Moreover, an architecture for normative systems, which contains subsystems for conditional obligations and permissions, is proposed in [25], bridging the gap between logical agent specification languages and agent architectures and programming languages.

Reo coordination language has also been applied to formal modelling long-running business transactions enabling the specification of complex compensation handling scenarios using a small number of modelling primitives [8,26,27]. Changizi et al. [28] introduced a unified toolset to formalise business process models including the Business Process Modelling Notation (BPMN), Unified Modelling Language (UML) Sequence Diagrams, and Business Process Execution Language (BPEL) in terms of Reo circuits. Recently, a framework for generating partially-distributed partially-centralised implementation of Reo connectors is proposed to support build-time compilation and run-time parallelism [29]. Other studies such as [30] merge both Reo networks and the Reo coordination tool to coordinate 2APL (A Practical Agent Programming Language) systems, thus focusing on the integration of Reo networks into the 2APL platform. Simulation has led to interesting animation tools such as the ECT plug-in for the Eclipse development environment [8,31,32]. This model checker tool supports code generation and graphical editing of the Reo connectors and constraint automata allowing animation, which demonstrates correctness of the formal model and assists designers in encountering scalability and usability limitations.

Methods like rCOS and Reo that are established based on a sound semantic theory can be used systematically for the design, automatic verification and coding of smart city systems with a toolkit. The significance is that the tool is also developed based on the sound theory of the model. Due to the aforementioned features, component based modelling and design, for instance, both rCOS and Reo methods, can deal with the complexity of software in cyber physical component systems and smart cities design, tackling the inclusion of complex analytics, modelling, optimisation, and visualisation [33,34,35].

## 3. System Description

**Figure 1:**
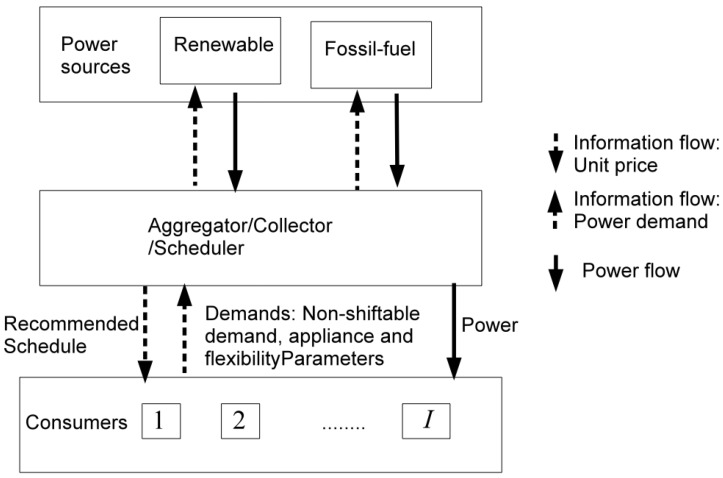
System model: roles and information flows.

With the installation of home area networks (HANs), smart meters and in-home display, consumers can not only monitor and manage the power consumption within the networked area that links thermostats, washing machines, clothes dryers and many more with a TV, PC and cell phone, but also interact with utilities and other consumers [36,37]. The proposed cooperative DR system as depicted in Figure 1 defines three possible *roles* that represent the concerned stakeholders namely:

- *Utility* is a set of energy suppliers shared by customers in a community. In this paper, we consider the utility as a combination of two energy suppliers: one is renewable energy supplier and the other is a fossil energy supplier.
- *Aggregator* is a centralised scheduler that compromises the plans from consumers and the available energy supply from the utility and finds out the “best” energy consumption strategy for the whole community, based on information about the available energy supply from the utility and the energy consumption plans from consumers.
- *Consumer* is a household equipped with a *smart meter* that is connected to the power line as well as the community network. A consumer may have some *household appliances* which function according to the supply that the Aggregator allocates to them.

These above roles will be interpreted as components with their own functionality in our architectural model. In addition, we see appliances of consumers also as components. The functionality of an appliance is not our concern however, because it is usually determined and defined by the producer.

### 3.1. Utility

The Utility makes essential information available to the consumers about both the reliable renewable and fossils, and energy supply planned for the upcoming 24 h.

**Definition 1** (Renewable and Fossil Energy Supply). *We denote by $P_{R,0}^{t}$ the energy supply generated from a set of renewable sources at a time slot* t∈{0,…,23}*. Similarly, $P_{F}^{t}$ represents the energy supply at time t generated from a set of fossil sources. The Utility centralizes the distribution of the energy, the notification to the Data Collector, and the billing process.*

### 3.2. Consumers

An ordered set *N* of consumers is willing to cooperate in the pursuit of global community targets (i.e., become greener), sending their data to the Aggregator. We consider a discrete *H*-hour time period, e.g., H=24 h, during which consumers schedule their electrical jobs [38].

**Assumption 1** (Consumer’s habits). Consumer habits, behaviours and use of appliances commonly demand a fixed energy load (formulae and benchmarks can be used to estimate appliance and home electronic energy use in kilowatt hours (kWh), as well as household local records—for example, refrigerator, alarm-controller, meters, standby televisions, water heater, etc.) as well as a variable load resulting from the utilisation of such appliances and other equipment or facilities.

**Assumption 2** (Home energy scheduler). An energy consumption scheduler (or home energy manager) connects via HAN, power-line communication (PLC) or any lower power wireless, such as ZigBee wireless standard [39], to all the appliances in the household.

The scheduler provides the consumer with an interface to allocate ”shiftable" demand at household-level, taking into account his/her time preferences.

Each consumer then pre-allocates a certain amount of fixed demand as well as variable consumption planned for the upcoming 24 h. Thus, consumer i∈N=1,2,…,N has a non shiftable demand of $x_{i,\mathrm{ns}}^{h}$ in timeslot *h*, h∈H representing the aggregated load of non-shiftable local consumption of their appliances and regarding frequent behaviours. Moreover, consumer *i* consists of shiftable appliances $A_{i}=\left\{a_{i,m},m\in 1,2,\ldots ,M\right\}$, where *M* is the total number of appliances of consumer *i* allowing shiftable consumption. Thus, $x_{i,\mathrm{sh}}^{h}$ denotes the variable energy consumption of consumer *i* in timeslot *h*.

### 3.3. Aggregator

**Assumption 3** (Data Aggregation). The Aggregator acts as a central node to carry out aggregation tasks, and communicates with the Utility as well as other Consumers.

The aggregator can obtain the available power supply information from renewable energy sources/providers and non-renewable sources. The available power supply from the renewable source is known for the upcoming *H* time-slots by the aggregator: $\left\{P_{R,0}^{1},P_{R,0}^{2},\ldots ,P_{R,0}^{H}\right\}$. The future smart grid highly emphasises the integration of distributed and renewable energy resources into the grid, together with the prioritised penetration of renewable energy for meeting the consumer demand. Thereby, in our model, the aggregator re-allocates a schedule to the consumers such that their demands are covered by the available renewable energy as far as possible, and the fossil fuel based energy is used only when necessary for the deficit power.

The above formulates our case study within the smart city system. Our cooperative DR system integrates a residential demand response strategy into utility planning taking into account the consumer comfort. The architecture along with its validation in Eclipse is presented in the following and accepts other smart city system compositions.

## 4. Cyber-Physical Components Modelling—rCPCS

The models of software components in rCOS [3,21] are extended with physical components that may be controlled by digital controllers. Refinement of Cyber-Physical Component Systems, namely rCPCS, is supporting the development of the proposed DR system.

### 4.1. State Variables, Interfaces and Interactions

In general, *cyber-physical component*, or simply “component” when there is no confusion, has *discrete state variables* that are directly changed by control programs, and *continuous state variables* whose changes follow differential equations, depending on states of the discrete variables. The state variables of a component *C*, denoted by αC, called the alphabet of *C*, is divided into two subsets, αC=〈βC,γC〉 of private discrete state variables and continuous state variables. For example, an appliance *A* of a household is a component with its state variables αA=〈{s:{on,off}},{rate:Real}〉, where rate is the rate in which energy is consumed by the appliance when it is in operation.

**Figure 2:**
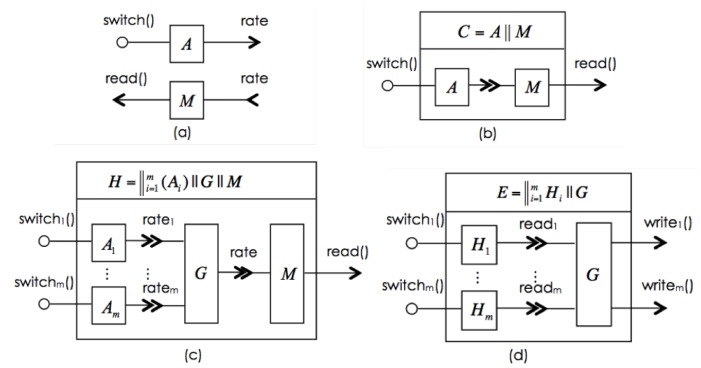
(**a**) *A*, representing an appliance, has a provide operation switch() and a provided signal rate. *M*, representing a meter, has a provide operation read(), and a required signal rate; and (**b**) A||M connects the provided signal of *A* and the required signal of *M*, thus forming a component that provides operations switch() and read(). Different ways of composing components represent different design approaches: (**c**) evolution of a household with a meter; and (**d**) evolution of an Aggregator.

The interfaces provide the means for the component to interact with its environment (i.e., other possible cyber-physical components including human actors). A component *C* can have a *provided interface* (or *input interface*), C.pIF, and or a *required interface* (or *output interface*), C.rIF; but a component must have an interface. Each of the interfaces contains two sets, C.pIF=〈pO,pW〉 of *provided operations* and *provided signals* or *wires*, or C.rIF=〈rO,rW〉 of *required operations* and *required signals*. It is required that the set of provided signals is a subset of the continuous variables of the component (i.e., pW⊆γC). The variables γC−pW are the *private continuous variables* of *C*. Figure 2a shows an appliance *A* with a provided interface A.IF=〈{switch()},{rate}〉 (i.e., one provided operation switch()).

Interactions can be performed with other digital or physical components. For example, a digital controller can be designed to interact with *A* to switch the appliance “on” and “off”, and a meter to record the energy consumption by using the rate. On the other hand, interactions of *A* can be with human actors, for example the householder can “observe” or use the rate to “calculate” the energy consumption and “switch” the appliance “on” and “off”. One can imagine the evolution from interactions of the appliance with human operators to interactions of digital controllers and meters. This would be one step of increase in automation, but the model of the functionality, behaviour, including interactions behaviour, of the appliance remains unchanged.

### 4.2. Local Functionality and Behaviour

The discrete variables change through execution of programme instructions that are enabled via invocations to interface operations. The execution of these operations is called *local functionality*. Note that the behaviour of the continuous variables is controlled through the change of the discrete variables. Therefore, the local functionality defines the behaviour or abstract semantics of the control programme.

Besides discrete functionality, cyber-physical components can also show continuous evolution for its continuous variables defined in time-dependent functions, often differential equations. For example, the continuous evolution for the appliance *A* can be defined by rate as the rate in which energy is consumed by the appliance when it is on, and the rate is assumed to 0 when the appliance is off. We believe the definition of rate is usually provided by the manufacture of the appliance. Thus, the *behaviour* of *A* is that the rate evolves along with the switches on and off of the component *A*. Consider, for instance, an electronic meter *M* that records the accumulated consumption of energy of appliance *A*. Its provided interface M.pIF comes up with a signal read and its required interface M.rIF consists of a single signal rate. The behaviour of *M* (i.e., the evolution of read) is a timed function of the required signal rate. For example, it can be defined as $read\left(t\right)=\int_{0}^{t}rate$. Therefore, in general, the behaviour of the continuous variables is defined by timed functions of the discrete variables and the required signals. In general, the continuous behaviour (or the trajectories) of the continuous variables of a component *C* is specified by timed functions of the following form, where feedbacks loops are possible γC=F(βC,γC,rW).

### 4.3. Component Composition

Components are composed through their interfaces. rCPCS interfaces include signals for the composition of physical components. Interfaces also bridge different technologies, whereas different ways of composing components represent different design approaches. For example, Figure 2b depicts a composite component C=A∥M composing appliance *A* and meter *M*. Thus, to design a household component as in Figure 2c, we consider an arbitrary number *m* of appliances in a household, each modelled by $A_{i}$ for i=1,…,m and meter *M*. A main switch connector, denoted by *G* centralises $rate_{i}$ interfaces such that it has a continuous variable rate as its provided interface and $\left\{rate_{i}\;|\;i=1,\ldots ,m\right\}$ as provided interfaces. The behaviour of *G* is $rate=\sum_{i=1}^{m}rate_{i}$. Thus, the household can be modelled by $H=(\left({∥}_{i=1}^{m}A_{i}\right)∥G)∥M$. Similarly, a switch connector *G* can also be used to summate the fixed and variable demands of individual households. Component *E* in Figure 2d represents a scheduler that reads $read_{i}$ from $M_{i}$ to decide when appliances can be switched on to operation according to the energy combustion budget ${\mathrm{fx}}_{i}$ and ${\mathrm{vx}}_{i}$.

The model of the Utility by a component *U* is simple. It only provides an operation request(x:Real,y:Real;z:Real) for the supply of energy. Its execution provides the amount of committed supply for the day through the return parameter. The *E* component has an interface (i.e., an active process), through which it periodically calls the interface operations $Rf_{i}\left(\right)$ and $Rv_{i}\left(\right)$ and makes a request to utility *U* via request(). Once it receives notification from *U* about the committed supply, it “negotiates” with the households and reallocates budgets, namely $Wf_{i}\left(\right)$ and $Wv_{i}\left(\right)$. Each household $H_{i}$ is then managed by its own. We represent this system scenario as E∥U.

### 4.4. Discussion

With rCPCS, we demonstrate the importance and effectiveness of building models of the system architecture with respect to the following aspects:

1. As the system grows larger, abstraction and decomposition in building the system architecture becomes essential for dealing with complexity.
2. The modelling method supports top-down development and bottom-up synthesis; more importantly, it supports both component-based system evolution and component-based incremental design model building.
3. The modelling method supports different implementations of coordination and control of components on different hardware platforms.
4. It supports tool development for simulation and verification.

rCPCS allows system evolution in different ways. For example, the composition ${∥}_{i=1}^{k}H_{i}$ of the households behaves exactly the same as one household in a “black box” if a connector is added to summate the fixed and variable demands of the individual households. This shows how abstract modelling deals with complexity. Similarly, we can imagine that a network of utilities works in collaboration to provide power supply. Once they reach agreement on how to share the supply upon the Aggregator’s request, they interface with the Aggregator as a single utility. Furthermore, the centralised Aggregator can be transformed into a distributed implementation such that the reallocation can be performed among households themselves. We can also envision a component home manager HM that allows the householder to set budgets ${\mathrm{fx}}_{i}$ and ${\mathrm{vx}}_{i}$ up for each appliance $A_{i}$; it then controls the operations of the appliance to meet budgets and calculates consumption $read_{i}$ of $A_{i}$ from its $rate_{i}$. It is also possible to arrange a distributed scheduling solution in which the control on $A_{i}$, along with ${\mathrm{fx}}_{i}$ and ${\mathrm{vx}}_{i}$, is embedded in meter $M_{i}$.

The architectural model is also important for identifying and analysing vulnerabilities and weaknesses within the different components due to interaction mechanisms, communication protocols, hardware quality or software bugs. Based on this hazard and risk analysis, architectural decisions can be made for different concerns, such as distribution, use of redundancy, specially designed secure protocols, etc., to improve safety, security, integrity, and availability.

Moving towards interoperable smart city systems, rCPCS is introduced as a formal framework for modelling and analysis of smart city systems and their compositions, making it easy for designers and planners to integrate platforms, devices and applications. Through the implementation of rCPCS in Reo Coordination Language and the Extensible Coordination Tools for the Eclipse platform, we especially focus on testing its adaptivity modelling aspects such as switching/extending the different components according to the varying requirements.

## 5. rCPCS Implementation in Reo

Tool support, such as simulation and verification, is important for rCPCS practical adoption. In this section, we model its component communication patterns by means of Reo connectors and the Eclipse ECT plug-in.

### 5.1. Basic Concepts

Reo [7,35,40,41] is a channel-based exogenous coordination model wherein complex coordinators, called *connectors*, are compositionally constructed from simpler ones. Components in Reo are computational entities that are able to store, manipulate produce and receive data and messages from other components. Components may be located at different places physically. A complex component may contain other components as part of it as well and are connected through *connectors*.

**Figure 3:**
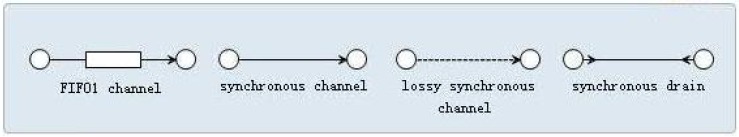
Some basic channels in Reo.

*Channels* in Reo have two types of channels ends: *source* and *sink*. A source channel end accepts data into its channel, and a sink channel end dispenses data out of its channel. It is possible for the ends of a channel to be both sinks or both sources. Reo places no restriction on the behaviour of a channel and thus allows an open-ended set of different channel types to be used simultaneously. Each channel end can be connected to at most one component instance at any given time. Figure 3 shows the graphic representation of some basic channels:

- *FIFO1 channel* represents an asynchronous channel with one buffer cell which is empty if no data item is shown in the box. If a datum *d* is contained in the buffer of a FIFO1 channel, then *d* is shown inside the box in its graphical representation.
- *Synchronous channel* has a source and a sink end and no buffer. It accepts a data item through its source end when it can simultaneously dispense it through its sink.
- *Lossy synchronous channel* is similar to a synchronous channel except that it is able to accept data through its source end at all times. The datum is transferred if it is possible to dispense it through the sink end; otherwise, the datum is lost.
- *Filter* with a pattern *P* specifies the type of data that are permitted to transfer through it. Any datum that conforms to the pattern is accepted through the source end when at the same time the sink end is ready to dispense it. Any datum that does not conform to the pattern is still accepted through the source end but will be immediately discarded.
- *Synchronous drain* is similar to a synchronous channel except that it has two source ends and no sink ends. It consumes a pair of data from its two source ends simultaneously and discards them away.

Complex connectors are constructed by composing simpler ones mainly via the *join* and *hiding* operations. Channels are joined together in a node that consists of a set of channel ends. Nodes are categorised into *source*, *sink* and *mixed nodes*, depending on whether all channel ends that coincide on a node are source ends, sink ends or a combination of the two. The hiding operation is used to hide the internal topology of a component connector. The hidden nodes can no longer be accessed or observed from outside. A complex connector has a graphical representation, called a *Reo circuit*, a finite graph where the *nodes* are labeled with pair-wise disjoint, non-empty sets of channel ends, and the *edges* represent the connecting channels. The behaviour of a Reo circuit is formalised by means of the data flows at its sink and source nodes. Intuitively, the source nodes of a circuit are analogous to the input ports, and the sink nodes to the output ports of a component, while mixed nodes are its hidden internal details. Components cannot connect to, read from, or write to mixed nodes. Instead, data-flow through mixed nodes is totally specified by the circuits they belong to.

A component can write data items to a source node that it is connected to. The write operation succeeds only if all (source) channel ends coincident on the node accept the data item, in which case the data item is simultaneously written to every source end coincident on the node. A source node, thus, acts as a replicator. A component can obtain data items, by an input operation, from a sink node that it is connected to. A take operation succeeds only if at least one of the (sink) channel ends coincident on the node offers a suitable data item; if more than one coincident channel end offers suitable data items, one is selected nondeterministically. A sink node, thus, acts as a nondeterministic merger. A mixed node nondeterministically selects and takes a suitable data item offered by one of its coincident sink channel ends and replicates it into all of its coincident source channel ends. A component can not connect to, take from, or write to mixed nodes.

### 5.2. Reo Circuits

**Figure 4:**
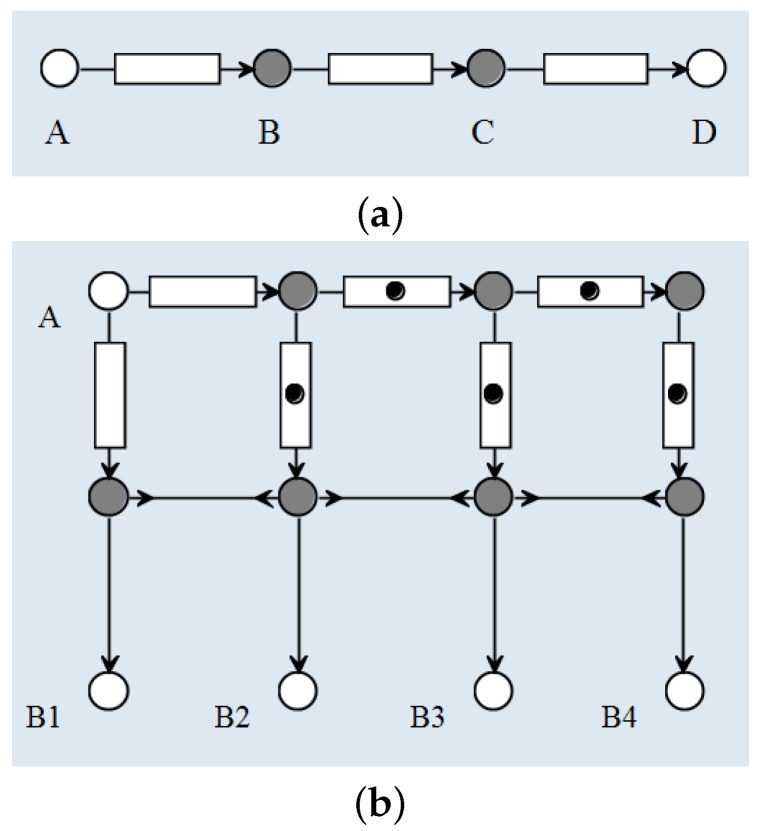
Examples of some Reo circuits. (**a**) a FIFO3 channel, constructed from some FIFO1 channels, gets data from its input node, temporarily stores it in an internal buffer of size 3, and propagates it to its output node); and (**b**) a Gatherer4 channel constructed from FIFO1 channels, synchronous channels and synchronous drains.

**Example 1** (FIFO connectors). *A FIFOn connector is similar to a FIFO1 channel except that it has a buffer with a capacity of n instead of* 1*. It consists of a chain of n FIFO1 channels. Figure 4a shows an example of a FIFO3 channel that is constructed from three FIFO channels: AB, BC and CD. The grey nodes (B and C) are mixed nodes while the white ones are either source node (as node A) or sink node (as node D).*

Initially, the three buffers (shown as blank boxes) are empty. Once a datum arrives at node A, it is accepted and stays in the left buffer. When the datum is staying in the left buffer, the FIFO1 channel AB is full and cannot accept any new data through node A. In the meantime, this datum is going to be transferred automatically from the left buffer to the middle buffer through the mixed node B because the middle buffer is empty. After that, the FIFO3 channel is able to accept another datum from A and store it in the left buffer.

**Example 2** (Gatherer connectors). A Gatherern connector consists of one source node and n sink nodes. A Gatherern connector is similar to a FIFOn channel such that they are able to store the data previously accepted through the source node as long as the memory does not run out. The difference lies in the fact that FIFOn channel distributes one piece of data at a time while a Gatherern connector dispenses (but does not discard) all the data in its memory. Every time a Gatherern connector dispenses, it only discards the oldest datum in its memory and reserves the vacant space for a new datum.

Figure 4b depicts a Gatherer4 connector on its initialisation with some FIFO1 channels full (shown as a dot in the box). It has a source node A for inputs and four sink nodes $B_{1}$ ... $B_{4}$ for outputs. Node $B_{1}$ always delivers the latest datum in the memory, whereas $B_{2}$ dispenses the second latest datum, and so on. The four sink nodes deliver the data at the same time, which is guaranteed by the synchronous drains between the set of four mixed nodes in the middle.

Once the connector is initially set-up, it is ready for the first datum arriving into node A. After the first datum, which is also the latest datum in the memory, data will be distributed through node $B_{1}$. However, since there is no second (and third and fourth) data yet, there is nothing that can be delivered from node $B_{2}$ (and $B_{3}$ and $B_{4}$). Therefore, when the Gatherern connector is initialised, it needs some redundant data in its memory, as the full FIFO1 channels shown in Figure 4b.

### 5.3. Utility–Aggregator Communication

The utility sends the amount of available renewable resources $P_{R,0}^{1},\ldots ,P_{R,0}^{H}$ and fossil fuel resources $P_{F}^{1},\ldots ,P_{F}^{H}$ to the aggregator, so the utility–aggregator communication is a one-way communication. In a more complex context that considers billing, we may ask the utility to send the price information to the aggregator who returns billing information about each consumer back to the utility. In that case, the utility–aggregator communication becomes a two-way communication. Furthermore, the utility and the aggregator may be physically located at different places far from each other, so the communication between them cannot be done in a flash. In that case, we may add some timing properties on the communication. At any case, Reo connectors are sufficient to model the communication behaviours [41].

**Figure 5:**
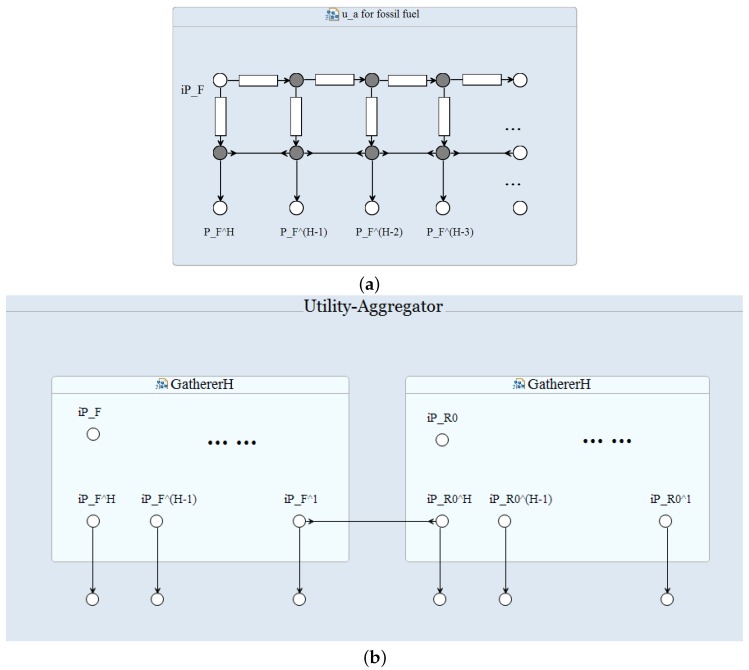
Utility–aggregator communication. (**a**) a Gatherer*H* connector that is used to construct a communication pattern between the utility and the aggregator; and (**b**) the utility–aggregator connector.

In practice, the aggregator schedules the energy consumption round by round. At every round, the utility is responsible to offer the *latest* fossil fuel resources $iP_{F}$ (and renewable resources resp.), and the aggregator should be able to collect the fossil fuel resources limits for the past *H* hours $P_{F}^{H},\ldots ,P_{F}^{1}$. Therefore, we have to provide a mechanism to store the utility supply generated in the past *H* hours and provide the aggregator with this information at a time through the nodes $P_{F}^{H}$, $P_{F}^{H-1}$, ..., $P_{F}^{1}$. Gatherer*H* connector supports this functionality (Figure 5a). At every round, the utility writes the latest fossil fuel supply through node $ip_{F}$, and the aggregator collects this load for the past *H* hours from node $P_{F}^{H}$, $P_{F}^{H-1}$, ..., $P_{F}^{1}$. In a similar way, we can construct a gatherer connector for the supply available from renewable resources. The entire communication pattern between the utility and aggregator is shown in Figure 5b.

*A note on the Aggregator’s local functionality.* The Aggregator holds the following algorithmic statements according to the available supply from renewable resources, as follows:

**Case 1:**

(1) $$\sum_{i\in N}x_{i,\mathrm{ns}}^{h}\geq P_{R,0}^{h}.$$

If Equation (1) is the case ∀h∈H or for most *h*, the renewable energy supply is not able to cover even the non-shiftable appliances, which means that the rest of the power partly for the non-shiftable appliances and wholly for the shiftable appliances should be supplied by the fossil fuel based source. The problem thus reduces to conventional shiftable-appliance-scheduling problem, where scheduling should be done for $\left(\sum_{i\in N}x_{i,\mathrm{ns}}^{h}-P_{R,0}^{h}\right)+\sum_{i\in N}x_{i,\mathrm{sh}}^{h}$:

**Case 2:**

(2) $$\sum_{i\in N}x_{i,\mathrm{ns}}^{h}<P_{R,0}^{h}.$$

In this case, in addition to satisfying the power requirements for non-shiftable appliances of all consumers, the renewable energy serves to supply shiftable appliances.

### 5.4. Consumer–Aggregator Communication

**Figure 6:**
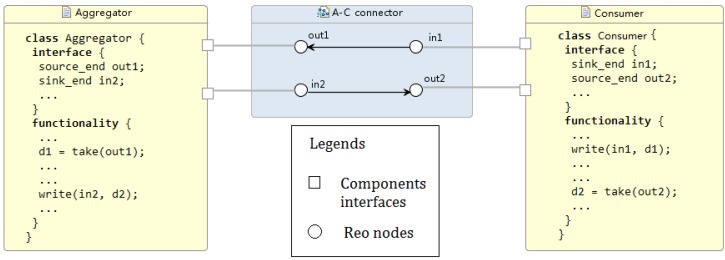
Consumer–aggregator communication architecture in Reo.

A consumer sends his power demand for the next few hours to the aggregator. Once the aggregator works out the power scheduling solution, it will send the result back to the consumer. Therefore, the consumer–aggregator communication is a two-way communication (Figure 6).

*A note on the Consumer’s local functionality.* Let us write the demand of Consumer *i* in timeslot *h* as $x_{i}^{h}=x_{i,\mathrm{ns}}^{h}+x_{i,\mathrm{sh}}^{h}$ for h∈H, where $\left\{x_{i,\mathrm{sh}}^{h}\right\}$ should be optimised. The shiftable appliances of consumer *i* specify the following parameters: $a_{i,m}:\left\{s_{i,m},e_{i,m},d_{i,m},c_{i,m}\right\}$, which represent the earliest possible starting timeslot, the last acceptable starting timeslot, the duration of operation in timeslots and the power consumption, respectively, of appliance $a_{i,m}$. Let $\gamma_{i}\in \left[0\;1\right]$ denote the flexibility factor of consumer *i*, where $\gamma_{i}=0$ indicates that the consumer prefers to operate its shiftable appliances in the earliest possible timeslots, and $\gamma_{i}=1$ implies that the consumer is totally flexible and does not mind operating its shiftable appliances in the last possible slots.

Let us define $P_{R}^{h}:=P_{R,0}^{h}-\sum_{i\in N}x_{i,\mathrm{ns}}^{h}>0,\;\forall h\in H$ according to Equation (2). Note that even if $\sum_{i\in N}\sum_{a_{i,m}\in A_{i}}{\left[c_{i,m}\right]}_{h=s_{i,m}}^{s_{i,m}+d_{i,m}}\leq \sum_{h\in H}P_{R}^{h}$ is true, $\sum_{i\in N}x_{i,sh}^{h}\leq P_{R}^{h}\;\forall h\in H$ may not hold. The goal of the aggregator can be achieved in two stages:

**Stage 1:**

(3) $$\min_{\left\{x_{i,\mathrm{sh}}^{h},\forall i\in N\right\}}P_{R}^{h}-\sum_{i\in N}x_{i,\mathrm{sh}}^{h},$$

(4) $$s.t.\;\sum_{i\in N}x_{i,\mathrm{sh}}^{h}\leq P_{R}^{h};\;s_{i,m}\leq s_{i,m}^{*}\leq e_{i,m}.$$

The solution to Equations (3) and (4) is $x_{i,\mathrm{sh}}^{*h}$ corresponding to the optimal starting timeslots $s_{i,m}^{*}$ for the appliances of consumers $i\in N_{1}$, where $I_{1}\subseteq N$.

**Stage 2:**

Let $N_{2}$ denote the set of consumers with appliances that could not be scheduled by solving Equations (3) and (4) such that $N_{1}\cup N_{2}=N$. The deficit power requirement is obtained from the fossil fuel source. For the fossil fuel source, the objective is to minimise the peak-to-average ratio (PAR) of supply from the source. Suppose the set of the appliances not scheduled in stage 1 be $A_{i}^{{}^{\prime }}$ for consumer *i*, $i\in N_{2}$. Let $x_{i,\mathrm{sh}}^{{}^{\prime }h}$ be the shiftable load of consumer *i*, $i\in N_{2}$ in timeslot *h*. Thus, the power requirement from the fossil fuel source will be $x_{i,\mathrm{sh}}^{{}^{\prime }h}\;\forall h\in H,i\in N_{2}$ and $a_{i,m}\in A_{i}^{{}^{\prime }}$. The objective of the aggregator in this stage is therefore:

(5) $$\min_{\left\{x_{i,\mathrm{sh}}^{{}^{\prime }h},\forall i\in N_{2}\right\}}\frac{\max\left(\sum_{i\in N_{2}}x_{i,\mathrm{sh}}^{{}^{\prime }h}-\left\{P_{R}^{h}-\sum_{i\in N_{1}}x_{i,\mathrm{sh}}^{*h}\right\}\right)}{\frac{1}{H}\left(\sum_{H\in H}\sum_{i\in N_{2}}x_{i,\mathrm{sh}}^{{}^{\prime }h}-\left\{P_{R}^{h}-\sum_{i\in N_{1}}x_{i,\mathrm{sh}}^{*h}\right\}\right)},$$

(6) $$s.t.\;\sum_{i\in N_{2}}x_{i,\mathrm{sh}}^{{}^{\prime }h}\leq P_{F}^{h};\;s_{i,m}\leq st_{i,m}\leq e_{i,m},$$

where $P_{F}^{h}$ represents the power generation limit of the fossil fuel source in timeslot *h*.

The solution to the optimal power allocation problem is $\left\{s_{i,m}^{*}\right\}$ obtained by solving Equations (3)–(6).

Since the overall problem is non-deterministic polynomial-time hard (NP-hard), existing work proposes approximations using well- known metaheuristic policies such as simulated annealing or some others based on linear programming [42] that provide accurate solutions in a fraction of the time required by the system. Though we will not investigate how these techniques would perform, the optimal consumption for consumer *i* that the aggregator has to obtain ∀i∈N is

(7) $$x_{i,\mathrm{sh}}^{*}=\sum_{a_{i,m}\in A_{i}}{\left[c_{i,m}\right]}_{h=s_{i,m}^{*}}^{s_{i,m}^{*}+d_{i,m}},$$

where $s_{i,m}^{*}\in \left[s_{i,m}\;\;\;e_{i,m}\right]$.

### 5.5. Consumer–Appliance Communication

**Figure 7:**
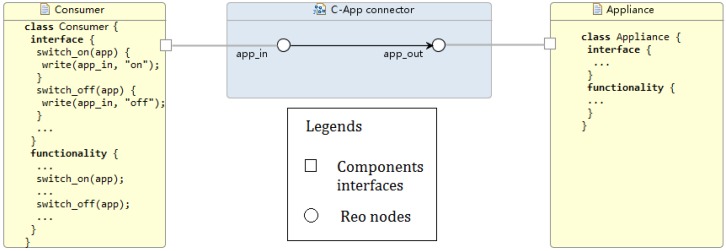
Consumer–appliance communication architecture in Reo.

A consumer can control his appliances through his home network. For each appliance, the consumer–appliance communication is a one-way communication (Figure 7).

*Algorithms in the Appliance Component.* Current smart home energy displays show instantaneous usage, expenditure and historic feedback as a minimum [43]. These displays can allow the consumer to coordinate the interaction among the devices participating in the scheduling. Its implementation should help user engagement and support the novel functionality. To schedule shiftable appliances, the home scheduler (class Consumer) provides consumer *i* with the interface to specify the following parameters $\forall a_{i,m}$: the earliest possible starting timeslot $s_{i,m}$, the last acceptable starting timeslot $e_{i,m}$, and the duration of operation in timeslots $d_{i,m}$. Moreover, consumer *i* can indicate a flexibility factor $\gamma_{i}\in \left[0\cdots 1\right]$ expressing his/her tolerance for operating the shiftable appliances at the earliest/last possible timeslots, respectively. Functionality procedure in class Consumer parametrised these elements through the user interaction. On the other hand, non-shiftable appliances consumption scheduling is automatically allocated by the home scheduler given the hourly usage patterns recorded by the smart meter. Once receiving the aggregator re-allocation, the Consumer scheduler shows the user how much shiftable load was removed or re-allocated. Appliances will automatically operate accordingly.

### 5.6. Experimental Results

**Figure 8:**
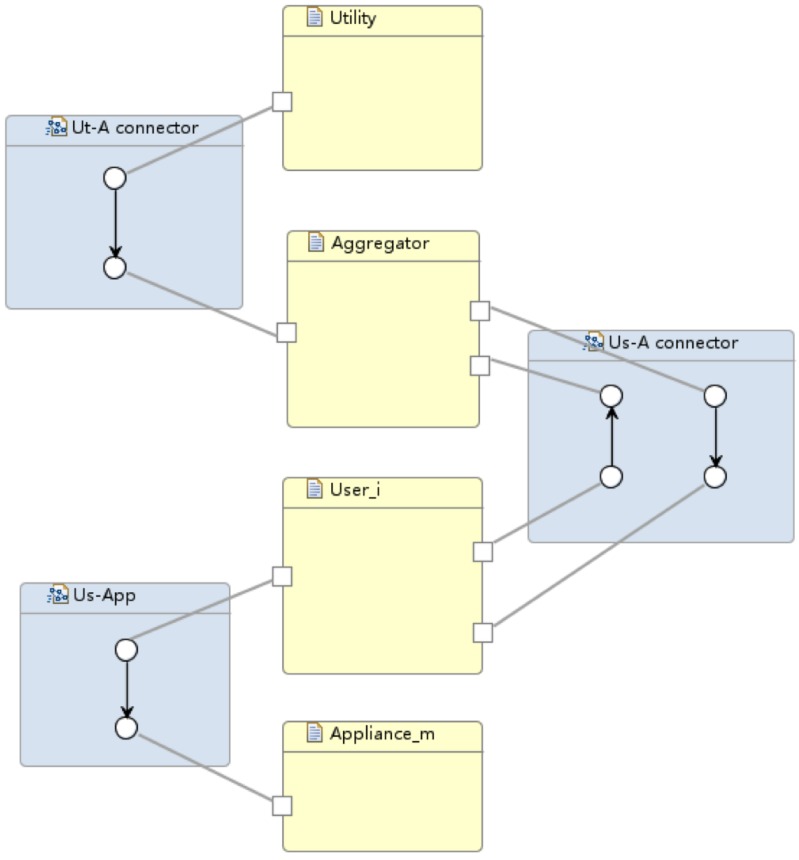
Cooperative demand response system architecture that allows scalability and system composition and coordination through separate connectors.

Our model consists of three Reo connectors (shown as blue boxes in Figure 8) that form the main body of the architecture. Connector animations of the Reo circuit on the ECT Eclipse confirm the correct communication of the system components. In particular, the following information flows are supported within the current implementation:

1. Utility–Aggregator: Connector *GathererH* consists of one source node and 24 sink nodes, i.e., the number of hours/slots which utility supply information is provided in. As described is Section 5.3, boxes are one space buffer and save up to one data item; as new data comes into the connector, boxes are filled up so the connector can save up to 24 data items. Two *GathererH* connectors serve the Aggregator to gather up both renewable and fossil available supply in kilowatt hour from the Utility; hence, every hour the input will be the updated iPR and iPF, respectively. Refined models might add a *GathererH* connector from Aggregator to Utility informing the latter about the reallocated supply vector for billing purposes and/or energy management.
2. Aggregator–Consumer: The *A-C connector* links the aggregator and a consumer with two simple synchronous channels. A synchronous channel with a source end “in” and a sink end “out” works as the assignment statement of out := in, transferring data (namely, consumers’ demand structure and allocated supply vector) between the aggregator and a consumer in a flash. However, in practice it is not always feasible because the aggregator and the consumer might be located in different places geographically. The transfer might take several microseconds or longer to proceed, and we need a mechanism to report to the sender whenever the connector fails to successfully transfer the data in time. Synchronous channels can be replaced by more delicate compound circuits made of lossy channels and timed channels. For instance, a timed channel is able to capture the behaviour of a monitor detecting whether the data is transferred successfully within a time threshold. Similarly, a lossy channel can capture data loss during network transmission [7,35].
3. Consumer–Appliances: *C-App connector* adopts similar semantics to the *A-C connector* but in a one-way communication shape. For more domotics features on appliances, we might need a two-way communication, thus including more functionality from Appliance towards Consumer. More complex connectors such as Feedback Loop, Sequencer and Inhibitor can be applied for appliances self-scheduling the available supply.

Each Reo connector has its own well-documented and/or formal specification (refer to [44] for a Reo connector repository), which makes reusability highly probable and preferable. As long as some standards are established for the protocols lying in between Utility, Users, Appliances and Aggregator, one can develop Reo connectors based on those standards and will be assured that the connectors fit in any implemented protocol.

## 6. Discussion

The formal model of the proposed architecture aims at making the design rigorously verifiable, enabling systematic change of architecture design through refinement or equivalence (that is, the algebraic equations proved in refinement calculus), and also making the design process repeatable. The correctness is guaranteed by the soundness of the semantic theory. An actual architecture of a given system implementation is proved by using the toolkit for model checking. Functional correctness is also guaranteed through model transformations implemented in the tool that are proven in the refinement calculus to preserve functional correctness. These models are at different levels of abstraction from the model of requirements through those of architecture design to code generation.

### 6.1. Model Checking

The algebraic laws established for refinement and abstraction assure scalability. There are an arbitrary number of components of the same type, such as a community of households or a large number of smarter meters that are treated as a single component, i.e., a single household, at a higher level (or integration) design of the system. Interoperability means the adaptability or customisability of components in different local contexts, and this is supported by changing interfaces through connector and coordinator components. Regarding this, we have shown how Reo connectors can specify the communication between the system components. A whole picture of the entire system architecture can be easily drawn from Figure 5a, Figure 6 and Figure 7.

Our architecture model has many advantages as follows:

- *Scalable.* Our model is scalable in that it supports an arbitrary number of consumers and appliances in the energy management system. If the communication patterns between components do not change, the architecture of the entire system will remain the same.
- *Component-Based.* Our model is a component-based approach. Different components can be developed independently because the interfaces and communication patterns are specified and determined in advanced by Reo connectors. Components can be implemented in any way as long as they respect the interfaces specified by the architecture model. As we will see, the aggregator can use any (centralised) scheduling algorithm in the architecture model and the entire system’s functionality will not change at all.
- *Practical.* Our model is not only a theoretical model, but also one that can automatically generate codes. Reo comes with a set of development tools including a Java code generator, which generates code for circuits based on their constraint automaton semantics. Therefore, all connectors (shown as blue boxes) are actually passages of Java codes that work as “glue codes” that connect all the components in the system.

Both the rCPCS formal model and its implementation in Reo with Eclipse guarantee that the system is correct-by-construction. Services’ (or systems’) composition are then possible as independent distributed entities that utilise Reo channels and connectors to communicate. Consider, for instance, our cooperative DR system efficiently accommodating plug-in hybrid electric vehicle (PHEV) fleets and making the EV penetration invisible to the system. The Aggregator represents a key component in this composition, which accepts reading real-time demand information from the passing-by vehicles via the control from a new connector. The connector will add PHEV demand to the Aggregator just like from a consumer, so nothing will change within its functionality. The Aggregator will keep notifying and updating consumers/PHEVs of the up-to-date scheduling vectors.

Finally, development and maintenance of computer programs certainly benefit from tool support, providing separation of concerns in the system design stages [45]. This represents the main motivation for using component-based architectures in the smart cities system design and integration, and validating/testing the architectures by Reo implementations. In this regard, Reo language is one of the Turing-complete models such as nondeterministic state machines and regular grammars, with expressive power to represent systems’ properties. Furthermore, the framework provided by Eclipse ECT certainly helps to empirically prove the property representations.

### 6.2. A Note on the Distributed Approach

Modern grid initiatives are moving toward distributed approaches that add much more autonomy, flexibility and scalability [46]. The use of multi-agent systems has become an increasingly powerful tool for energy management problems [47]. Typical objectives range from reducing peak power demand, utility energy costs and consumer bills [48], balancing energy supply and demand as well as improving grid efficiency [49], and increasing the share of renewable energy sources [50]. These can be achieved using a number of optimisation techniques such as integer, quadratic, stochastic and dynamic programming and also evolutionary algorithms [49].

We have identified two main approaches to the distribution and decentralisation of the scheduling task performed by the Aggregator. In any case, we can assume that consumers have complete knowledge of each others’ demands (anonymised) and the incoming *H*-period available renewable energy supply provided by the utility commercialiser. A game is then played by Consumers where they seek to minimise the global energy load per time-slot or, in other words, to satisfy Equation (2).

The simplest way is to make the Aggregator role take turns amongst the Consumers participating. The logic of the resource allocation algorithm would be exactly the same as when it runs on the Aggregator side.

A more complex and decentralised approach, based on the multi-agent architecture, consists of dividing the power management scheduling problem into subproblems involving different agents, each of which solves its own problem independently to find a solution to the whole problem. In general, this scheme seems to be scalable; however, it cannot guarantee obtaining the optimal solution or avoiding the complex interaction between the agents. Nevertheless, we found in [43], one of the most convenient techniques for the autonomous and distributed scheduling (which is a knapsack problem), namely, the backtracking algorithm. Backtracking incrementally builds a search tree that finds all feasible solutions, each of which is an energy consumption schedule represented by an M×N time table, where *N* is the number of tasks and *M* is the number of time-slots. Thus, each consumer knowing the others’ demands vectors and running the allocation procedure can satisfy a global peak reduction (or, in other words, achieve respective local peak reduction). Experiments in [43] show that when the number of tasks is over eight, a global coordination among scheduling units is desirable.

## 7. Conclusions

In this paper, we have proposed a scalable system architecture for a co-operative DR system, which is component-based, and proved component interoperability through formal modelling and languages. In the process of our component-based system design, we can be assured by the architectural description that the system under specification is correct by design with respect to the interoperability correctness of component cooperation. The proposed architecture, namely “Refinement of Cyber-Physical Component Systems” (rCPCS), extends the rCOS modelling method, and provides a powerful means of abstraction so that large and complex composite subsystems, such as the composition of all households, can be treated as as simple component. In addition, rCPCS architecture proposed for a DR system also supports different designs and different processes of system evolution as being integrated to a smart city system, in which new components with various interaction mechanisms can plugged into the existing system. By implementing rCPCS in Eclipse Extensible Coordination Tools and Reo language, we have proved scalability and correct interoperability amongst the heterogeneous system components. Reo has a very flexible infrastructure and can be applied to various application domains as a basis for test set design and decision-making. In our work, we successfully embedded smart grid and smart city systems in Reo’s framework, following the idea of Reo coordination middleware.

Our immediate future work includes the development of the formal semantics and algorithms presented in a Living Lab. We will also use the models to identify safety weaknesses and vulnerable components, as well as points about security threats in order to make architectural decisions to strengthen the system [51]. Furthermore, the introduction of intelligent components with learning capabilities is of interest for utilities and storage purposes.

## Abbreviations

The following abbreviations are used in this manuscript:

API	Application Programming Interface
DR	Demand Response
ECT	Eclipse Extensible Coordination Tools
HAN	Home Area network
IOP	Interoperability Open Platform
IoT	Internet of Things
MDPI	Multidisciplinary Digital Publishing Institute
PHEV	Plug-in Hybrid Electric Vehicle
PLC	Power-Line Communication
rCOS	Refinement Calculus for component and Object Systems
rCPCS	Cyber-Physical Component Systems

## References

[1] Palomar E., Liu Z., Bowen J.P., Zhang Y., Maharjan S. Component-based modelling for sustainable and scalable smart meter networks. Proceedings of the IEEE International Symposium on a World of Wireless, Mobile and Multimedia Networks (WoWMoM 2014), 3rd IoT-SoS Internet of Things Smart Objects and Service Workshop.

[2] Jifeng H., Li X., Liu Z. (2006). rCOS: A refinement calculus of object systems. Theor. Comput. Sci..

[3] Dong R., Faber J., Ke W., Liu Z. rCOS: Defining Meanings of Component-based Software Architectures. Proceedings of the ICTAC Training School on Software Engineering.

[4] Klein C., Kaefer G. (2008). From smart homes to smart cities: Opportunities and challenges from an industrial perspective. International Conference on Next Generation Wired/Wireless Networking.

[5] Tang S., Huang Q., Li X.Y., Wu D. Smoothing the energy consumption: Peak demand reduction in smart grid. Proceedings of the IEEE INFOCOM 2013.

[6] Mohsenian-Rad A.H., Wong V.W.S., Jatskevich J., Schober R. Optimal and autonomous incentive-based energy consumption scheduling algorithm for smart grid. Proceedings of the Innovative Smart Grid Technologies (ISGT).

[7] Arbab F. (2004). Reo: A channel-based coordination model for component composition. Math. Struct. Comput. Sci..

[8] Kokash N., Arbab F. (2013). Formal design and verification of long-running transactions with extensible coordination tools. IEEE Trans. Serv. Comput..

[9] Schaffers H., Komninos N., Pallot M., Trousse B., Nilsson M., Oliveira A. (2011). Smart Cities and the Future Internet: Towards Cooperation Frameworks for Open Innovation. The Future Internet.

[10] Blackstock M., Kaviani N., Lea R., Friday A. MAGIC Broker 2: An open and extensible platform for the Internet of Things. Proceedings of the Internet of Things (IOT).

[11] Filipponi L., Vitaletti A., Landi G., Memeo V., Laura G., Pucci P. Smart city: An event driven architecture for monitoring public spaces with heterogeneous sensors. Proceedings of the 2010 Fourth International Conference on Sensor Technologies and Applications (SENSORCOMM).

[12] da Silva W.M., Alvaro A., Tomas G.H., Afonso R.A., Dias K.L., Garcia V.C. Smart cities software architectures: A survey. Proceedings of the 28th Annual ACM Symposium on Applied Computing.

[13] Steuer S., Benabbas A., Kasrin N., Nicklas D. (2016). Challenges and Design Goals for an Architecture of a Privacy-preserving Smart City Lab. Datenbank-Spektrum.

[14] Papadopoulos G., Arbab F. (1998). Coordination models and languages. Adv. Comput..

[15] Garlan D., Monroe R.T., Wile D. (2000). Acme: Architectural description of component-based systems. Found Compon.-Based Syst..

[16] Edwards S., Lavagno L., Lee E., Sangiovanni-Vincentelli A. (2001). Design of embedded systems: Formal models, validation and synthesis. Readings in Hardware/Software Co-Design.

[17] Schutte S., Scherfke S., Troschel M. Mosaik: A framework for modular simulation of active components in smart grids. Proceedings of the 1st Internet workshop on Smart Grid modelling and simulation (SGMS).

[18] Pourvatan B., Sirjani M., Arbab F., Bonsangue M. Decomposition of constraint automata. Proceedings of the 7th international conference on Formal Aspects of Component Software (FACS).

[19] Chen X., He J., Liu Z., Zhan N. A model of component-based programming. Proceedings of the International Symposium on Fundamentals of Software Engineering (FSEN).

[20] Chen Z., Hannousse A.H., Van Hung D., Knoll I., Li X., Liu Z., Liu Y., Nan Q., Okika J.C., Ravn A.P. (2008). Modelling with relational calculus of object and component systems—rCOS. The Common Component Modeling Example.

[21] Chen Z., Liu Z., Ravn A.P., Stolz V., Zhan N. (2009). Refinement and verification in component-based model-driven design. Sci. Comput. Program..

[22] Diakov N.K., Arbab F., Diakov N., Arbab F. Compositional construction of web services using Reo. Proceedings of the International Workshop on Web Services: Modeling, Architecture and Infrastructure.

[23] Heydarnoori A., Mavaddat F., Arbab F. (2006). Towards an automated deployment planner for composition of web services as software components. Electron. Notes Theor. Comput. Sci..

[24] Zlatev Z., Diakov N., Pokraev S. (2004). Construction of negotiation protocols for E-Commerce applications. ACM SIGecom Exch..

[25] Boella G., van der Torre L. An architecture of a normative system: Counts-as conditionals, obligations and permissions. Proceedings of the Fifth International Joint Conference on Autonomous Agents and Multiagent Systems.

[26] Lazovik A., Arbab F. (2007). Using Reo for Service Coordination.

[27] Kokash N., Arbab F. Applying Reo to service coordination in long-running business transactions. Proceedings of the 2009 ACM Symposium on Applied Computing.

[28] Changizi B., Kokash N., Arbab F. A unified toolset for business process model formalization. Proceedings of the Formal Engineering Approaches to Software Components and Architectures.

[29] Jongmans S.S.T., Santini F., Arbab F. Partially-distributed coordination with Reo. Proceedings of the 2014 22nd Euromicro International Conference on Parallel, Distributed and Network-Based Processing (PDP).

[30] Arbab F., Aştefănoaei L., de Boer F.S., Dastani M., Meyer J.J., Tinnermeier N. (2008). Reo connectors as coordination artifacts in 2APL systems. Intelligent Agents and Multi-Agent Systems.

[31] Baier C., Klein J., Kluppelholz S. (2011). Modeling and verification of components and connectors. Formal Methods for Eternal Networked Software Systems (SFM).

[32] Clarke D., Costa D., Arbab F. (2007). Connector colouring I: Synchronisation and context dependency. Sci. Comput. Program..

[33] Lee E.A. Cyber physical systems: Design challenges. Proceedings of the 11th IEEE International Symposium on Object Oriented Real-Time Distributed Computing (ISORC).

[34] Xu T., Liu Z., Tang T., Zheng W., Zhao L. Component based design of fault tolerant devices in cyber physical system. Proceedings of the 2012 15th IEEE International Symposium on Object/Component/Service-Oriented Real-Time Distributed Computing Workshops (ISORCW).

[35] Chen X., Sun J., Sun M. A Hybrid Model for Connectors in Cyber-Physical Systems. Proceedings of the 16th International Conference of Formal Engineering Methods.

[36] Gellings C.W. (2009). The Smart Grid: Enabling Energy Efficiency and Demand Response.

[37] Darby S. (2010). Smart metering: What potential for householder engagement?. Build. Res. Inf..

[38] Myerson R.B. (1980). Conference structures and fair allocation rules. Int. J. Game Theor..

[39] Gungor V.C., Sahin D., Kocak T., Ergut S., Buccella C., Cecati C., Hancke G.P. (2011). Smart grid technologies: Communication technologies and standards. IEEE Trans. Ind. Inf..

[40] Baier C., Sirjani M., Arbab F., Rutten J. (2006). Modeling component connectors in Reo by constraint automata. Sci. Comput. Program..

[41] Arbab F., Baier C., de Boer F., Rutten J. (2007). Models and temporal logical specifications for timed component connectors. Softw. Syst. Model..

[42] Li G., Liu H. (2006). Resource allocation for OFDMA relay networks with fairness constraints. IEEE J. Sel. Areas Commun..

[43] Lee J., Kim H.J., Park G.L., Kang M. (2011). Energy consumption scheduler for demand response systems in the smart grid. J. Inf. Sci. Eng..

[44] Centrum Wiskunde & Informatica Institute, S.R.C. A Repository of Reo Connectors. http://reo.project.cwi.nl/webreo/.

[45] Fitzgerald J., Larsen P.G. (2009). Modelling Systems: Practical Tools and Techniques in Software Development.

[46] Pipattanasomporn M., Feroze H., Rahman S. Multi-agent systems in a distributed smart grid: Design and implementation. Proceedings of the 2009 IEEE/PES Power Systems Conference and Exposition.

[47] Bakr S., Cranefield S. Optimizing Shiftable Appliance Schedules across Residential Neighbourhoods for Lower Energy Costs and Fair Billing. Proceedings of the Joint Workshop Proceedings-AIH 2013/CARE 2013.

[48] Mohsenian-Rad A.H., Wong V.W.S., Jatskevich J., Schober R., Leon-Garcia A. (2010). Autonomous Demand-Side Management Based on Game-Theoretic Energy Consumption Scheduling for the Future Smart Grid. IEEE Trans. Smart Grid.

[49] Clement K., Haesen E., Driesen J. Coordinated charging of multiple plug-in hybrid electric vehicles in residential distribution grids. Proceedings of the 2009 IEEE/PES Power Systems Conference and Exposition.

[50] Banos R., Manzano-Agugliaro F., Montoya F., Gil C., Alcayde A., Gómez J. (2011). Optimization methods applied to renewable and sustainable energy: A review. Renew. Sustain. Energy Rev..

[51] Bowen J.P. (2000). The Ethics of Safety-Critical Systems. Commun. ACM.

